# Supplementary material for: Atlas of RNA editing events affecting protein expression in aged and Alzheimer’s disease human brain tissue
Source: Nat Commun. 2021 Dec 2;12:7035. doi: 10.1038/s41467-021-27204-9 (PMC8640037; doi:10.1038/s41467-021-27204-9)
Supplement: Supplementary file 1 — Supplementary information. [file 41467_2021_27204_MOESM1_ESM.pdf]

SUPPLEMENTARY MATERIALS

CONTENTS OF SUPPLEMENTARY MATERIAL:

Supplementary Data (excel sheet): Association results of those non-A-to-I editing events.

Fig. S1. Distributions of different types of RNA editing events across datasets.

Fig. S2. Tissue specificity of the known RNA editing events.

Fig. S3. Volcano plots of comparisons between different regions within each study.

Fig. S4. Differential expressions of *ADARs* across Alzheimer's disease (AD) status in ROSMAP unpaired 635 DLPFC samples.

Fig. S5. QQ plot of stage II meta-analyzed results and regional and forest plots at top loci.

Fig. S6. Distribution of frequent RNA recoding events across all datasets and their associations with AD status compared to the non-coding events.

Fig. S7. Functions of the principal components derived from the top AD associated editing events on the expressions of genes, isoforms and proteins.

Fig. S8. Mass-spectrometry figure of edited peptide (frequency ≥50%) hits in ROSMAP TMT proteomic dataset.

Table S1. Characteristic of the cohorts and picard metrics of RNA-seq datasets.

Table S2. The recoding RNA editing events with proteomic peptide hits.

Table S3. Regional differences in gene expressions of *ADARs*.

Table S4. Top RNA editing events associated with AD status.

Table S5. Association of *ORAI2* editing event (chr7:102096952, hg19) on multiple traits in ROSMAP unpaired DLPFC dataset (N=635).

Table S6. Comparisons between AD genes and non-AD genes.

Fig. S1. Distributions of different types of RNA editing events across datasets. Each dataset is presented as one stacked bar and different colors represent different types of editing events. On average, the majority of RNA editing events are the canonical A-to-I editing types, which are shown as the A-to-G and T-to-C editing types (≥90%) and the C-to-T and G-to-A types (5%).

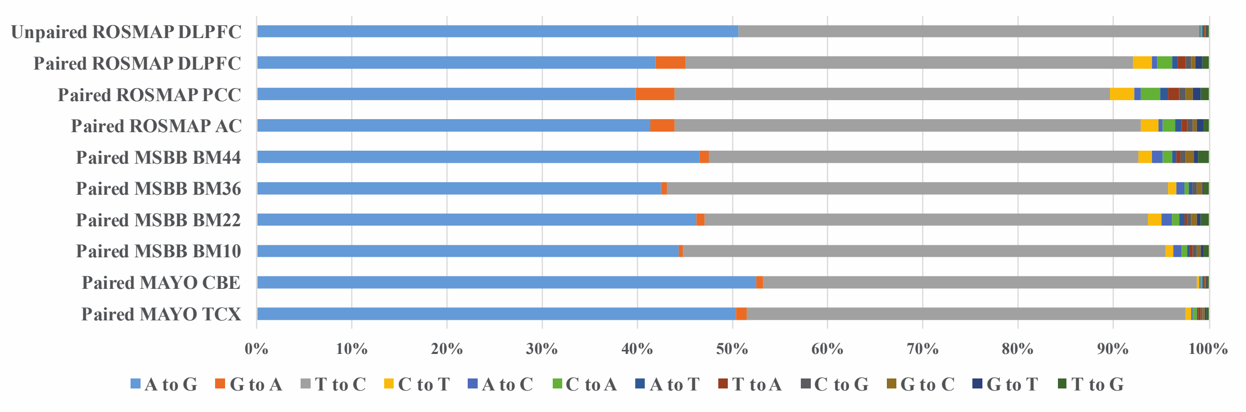

Fig. S2. Tissue specificity of the known RNA editing events. Pie chart showed the percentage of the known RNA editing events across different tissue types.

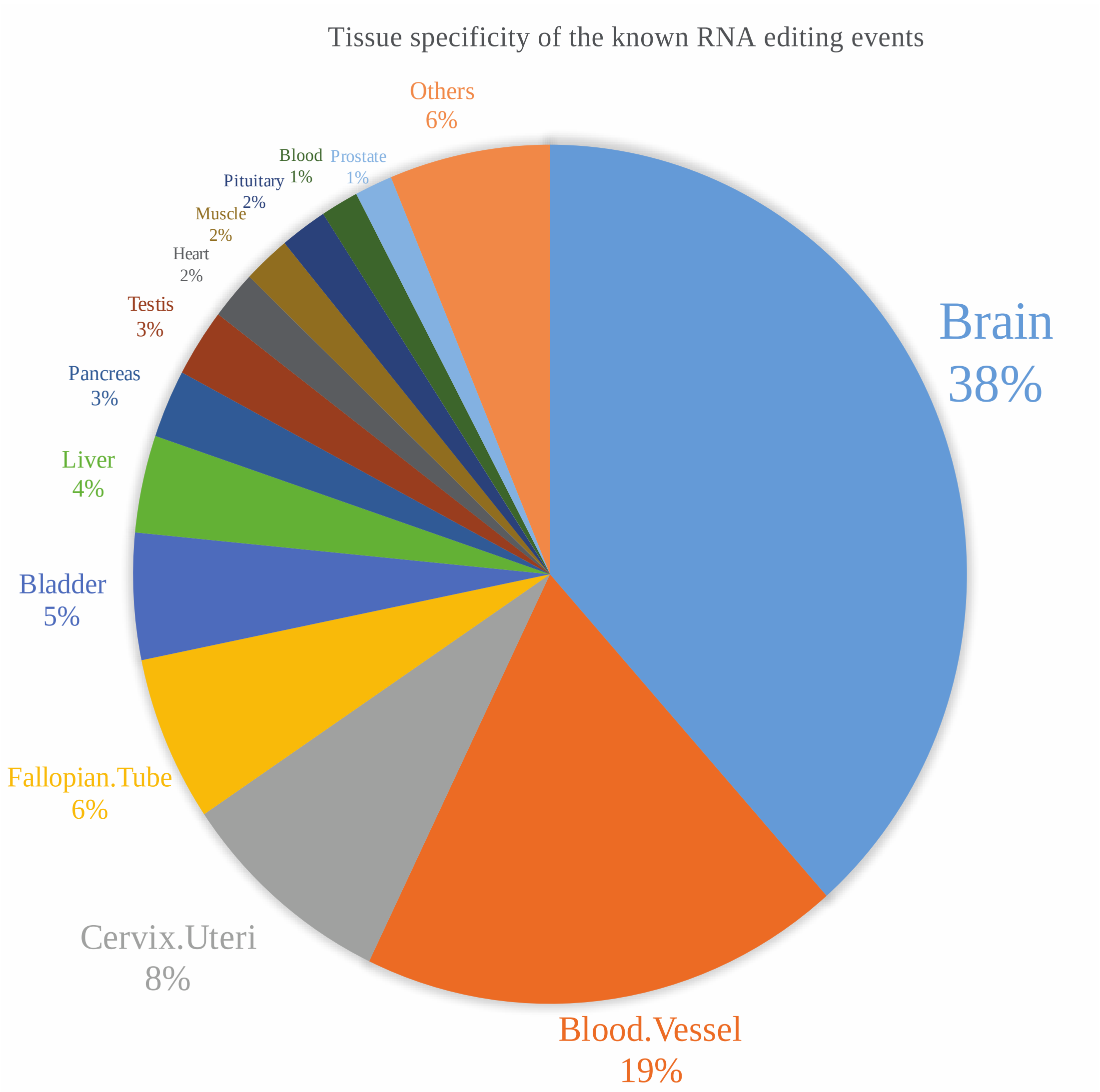

Fig. S3. Volcano plots of comparisons between different regions within each study. Comparisons of the level (% alternative reads) of each RNA editing event between 2 brain regions within each study were presented in a volcano plot. Each dot is one RNA editing event present in both brain regions. X and Y axes display the regression coefficient and its corresponding -log10 transformed *P* value of the exposure variable of 2 brain regions in the general linear mixed model which used subject as random effect, RNA editing level of each RNA editing event as dependent variable and its covariates include age at death, sex, postmortem interval (PMI), and RIN score using R lme package. The red horizontal line showed the Bonferroni-corrected P value threshold of 1.49×10^{-6} (0.05/33,641). The red dots with annotated gene names are those RNA editing event with $P \leq 1.49 \times 10^{-6}$ and regression coefficient of the differences between 2 brain regions ≥30%. X and Y axes display the regression coefficient and its corresponding -log10 transformed *P* value from the mixed linear regression model with the subject as random effect and fixed covariates of age at death, sex, postmortem interval and RIN score. The displayed *P* values were not adjusted by the multiple testings and were derived by two-sided tests.

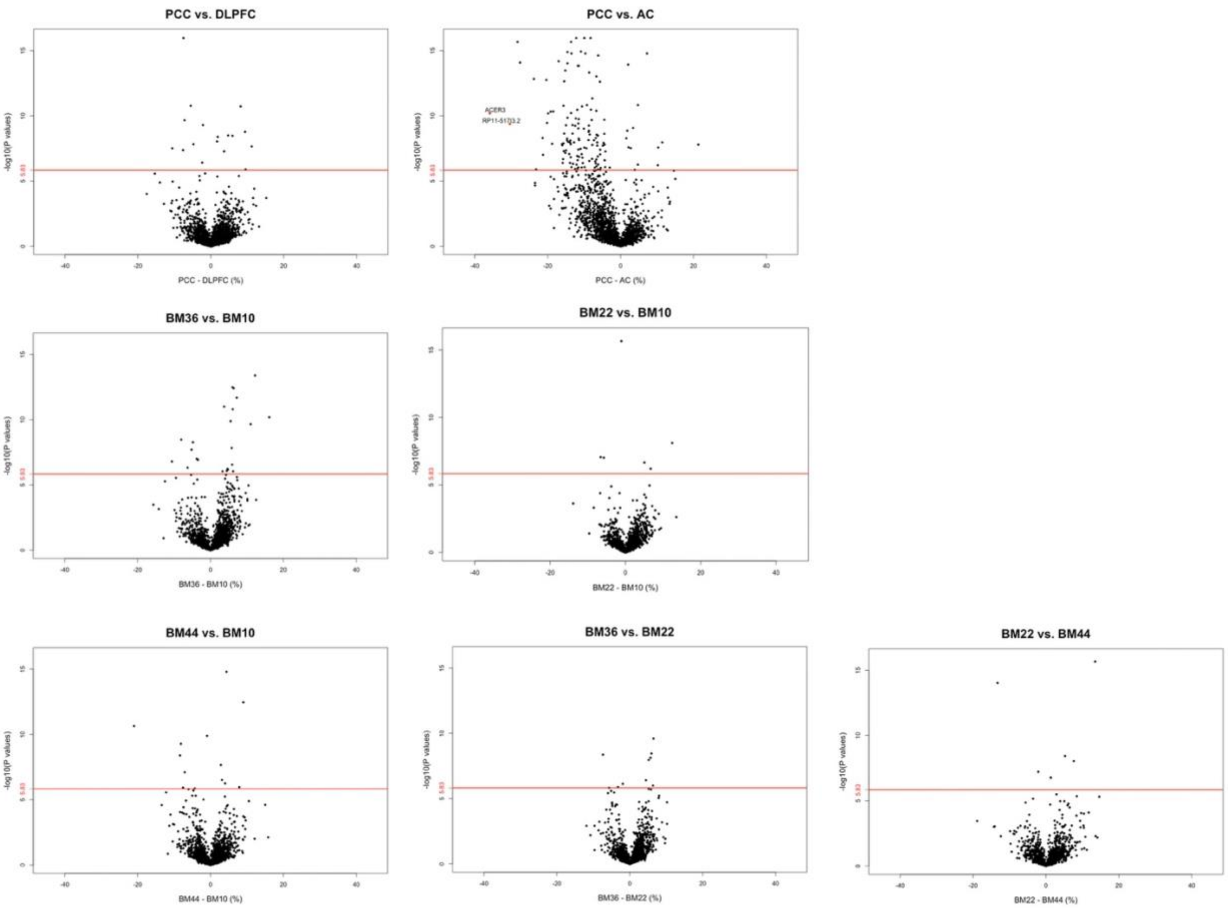
PCC vs. DLPFC
PCC - DLPFC (%)
PCC vs. AC
PCC - AC (%)
BM36 vs. BM10
BM36 - BM10 (%)
BM22 vs. BM10
BM22 - BM10 (%)
BM44 vs. BM10
BM44 - BM10 (%)
BM36 vs. BM22
BM36 - BM22 (%)
BM22 vs. BM44
BM22 - BM44 (%)
-log10(P values)

Fig. S4. Differential expressions of ADARs across Alzheimer's disease (AD) status in ROSMAP unpaired 635 DLPFC samples. All the panels in this figure have the same sample size (N=635 subjects). We showed the level of RNA expressions of (**a**) *ADAR1*, (**b**) *ADAR2*, (**c**) *ADAR3* and (**d**) the calculated value combining all three types of *ADARs* (*ADAR1+ADAR2-ADAR3*), based on their previously reported potential functions on RNA editings (*ADAR1* and *ADAR2* increase while *ADAR3* inhibits RNA editings), in subjects who cognitively non-impaired (NCI, blue), mildly impaired (MCI, pink), or fulfill criteria for AD dementia (AD, yellow). The RNA expression of *ADARs* (log2 transformed FPKM) are displayed by the notched boxplot, in which the lines from the bottom to the top represents the minimum value or the value <25% percentile, 25% percentile, median, 75% percentile, the maximum value or the value >75% percentile. The "Notch" around the median represent the 95% confidence interval of the median, and the area with color represent the interquartile with 50% of values. The open circle represents the potential outliers. The mean ADARs expressions are displayed in the legend box on the right top corner, in which the P value for the general linear regression model with ADARs expression as dependent variable and 3 AD status as exposure variable. The displayed *P* values were not adjusted by the multiple testings and were derived by two-sided tests.

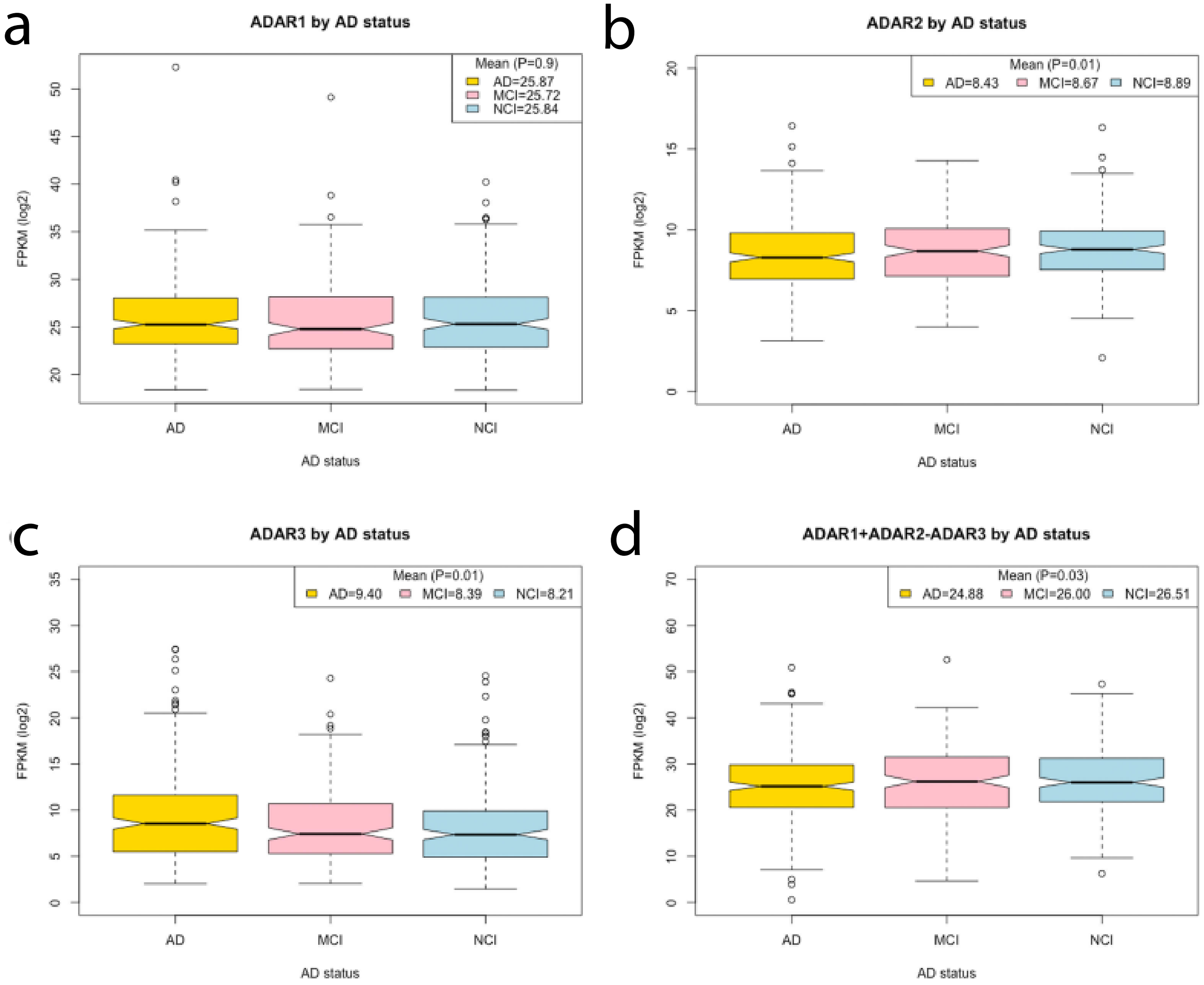
a
ADAR1 by AD status
Mean (P=0.9)
AD=25.87
MCI=25.72
NCI=25.84
FPKM (log2)
AD
MCI
NCI
AD status
b
ADAR2 by AD status
Mean (P=0.01)
AD=8.43
MCI=8.67
NCI=8.89
FPKM (log2)
AD
MCI
NCI
AD status
c
ADAR3 by AD status
Mean (P=0.01)
AD=9.40
MCI=8.39
NCI=8.21
FPKM (log2)
AD
MCI
NCI
AD status
d
ADAR1+ADAR2-ADAR3 by AD status
Mean (P=0.03)
AD=24.88
MCI=26.00
NCI=26.51
FPKM (log2)
AD
MCI
NCI
AD status

Fig. S5. QQ plot of stage II meta-analyzed results and regional and forest plots at top loci. (a) QQ plot. (b-e) Regional (upper) and forest (lower) plots of top loci at *SOD2* (b), *MCUR1* (c), *HSDL2* (d) and *PFKP* (e). Regional plot shows the Stage I association results (upper). Each circle represents a frequent RNA editing event with the top one in purple. The correlation (r) between the top one and the others are shown with the color coding of: shallow to dark red for $0<r\leq1$, white for $r=0$, and shallow to dark blue for $-1\leq r<0$. The correlation matrix between each pair is shown in a triangle (lower) with the same color coding. Forest plot show results in each dataset and Stage II meta-analyzed results. The estimated difference in the mean level of RNA editing (% alternative reads) by clinical AD status (0 for normal controls, 1 for mild cognitive impairment, and 2 for AD) and its 95% confidence interval were illustrated by the filled square and horizontal line for each dataset or the filled red diamonds for the summaries. The displayed *P* values were not adjusted by the multiple testings and were derived by two-sided tests.

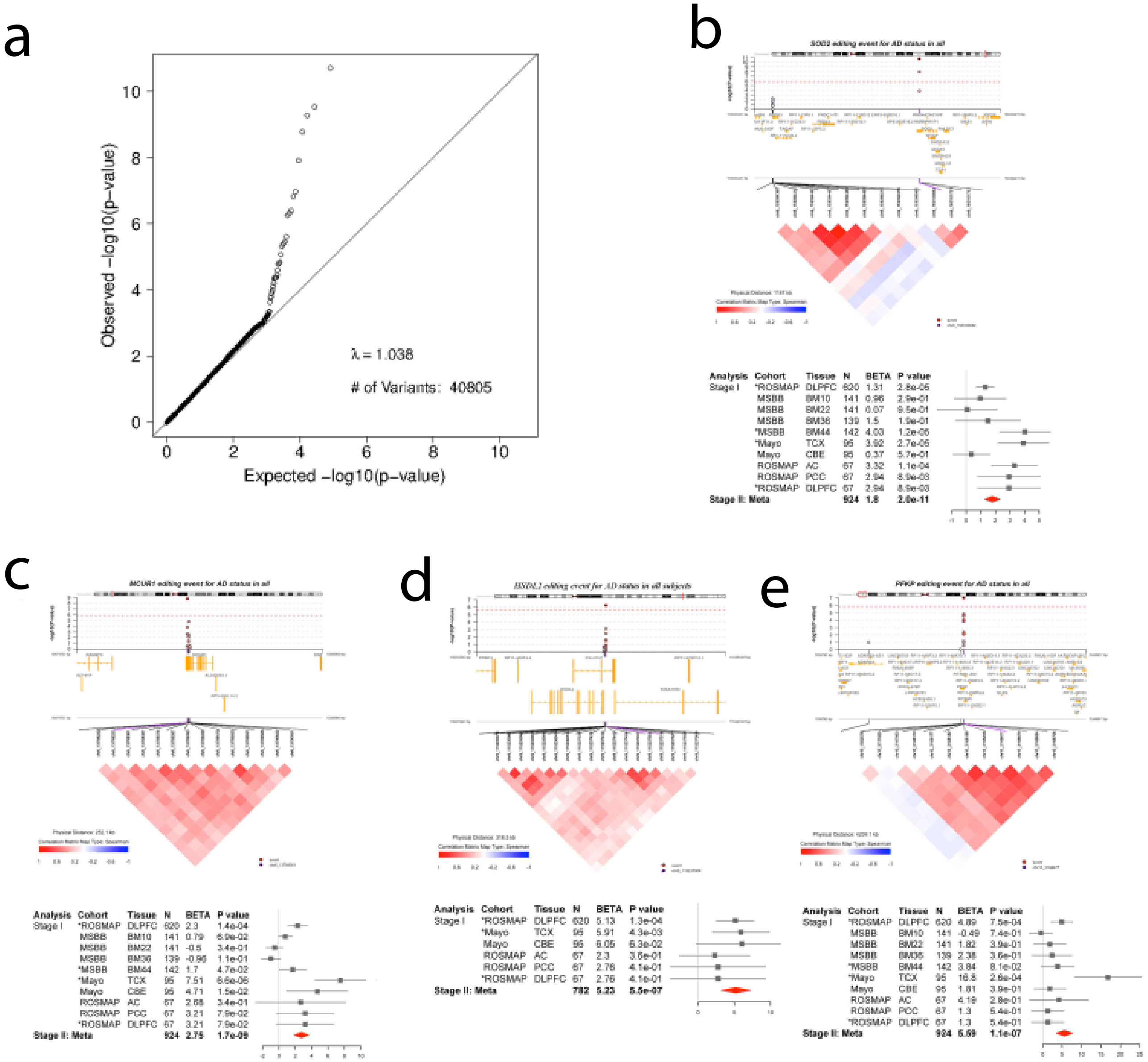
a
b
c
d
e
Observed −log10(p−value)
Expected −log10(p−value)
λ = 1.038
# of Variants: 40805

Fig. S6. Distribution of frequent RNA recoding events across all datasets and their associations with AD status compared to the non-coding events. (a), Percentage and number of RNA recoding events called in different RNA-seq datasets. The percentages are shown in blue bars and the number on each bar represent the counts of RNA recoding event. (b), Box plots (upper panel) and density plots (lower panel) showed the comparisons of the AD association *P* values (-log10) of the non-coding (gold) and re-coding events (blue) called across all different studies with RNA-seq datasets. The samples size (N) for each study are 635 subjects in the unpaired ROSMAP DLPFC dataset, 68 subjects in the paired ROSMAP datasets, 142 subjects in the paired MSBB datasets, and 229 subjects in the paired MAYO datasets. Y axis showed their meta-analyzed *P* values for the general linear model (glm) with RNA editing level (% alternative reads) as dependent variable, clinical AD status (0, 1, and 2 represent normal controls, mild cognitive impairment (MCI), and AD patients) as exposure variable, and covariates include sex, age at death, PMI, RIN, experimental batch, study (in 2 of the ROSMAP DLPFC datasets), race (in MSBB BM44 dataset), and tissue source (in MAYO temporal cortex dataset). The distributions of the P values by different types of RNA editing events across all datasets are represented by boxplots where the minimum and the maximum values are represented by the lowest and highest end of the vertical line passing the center of the box, and the first quartile (25%), the median (50%), the third quartile (75%) of the values are represented as the lower, center, and the higher horizontal lines of the box.

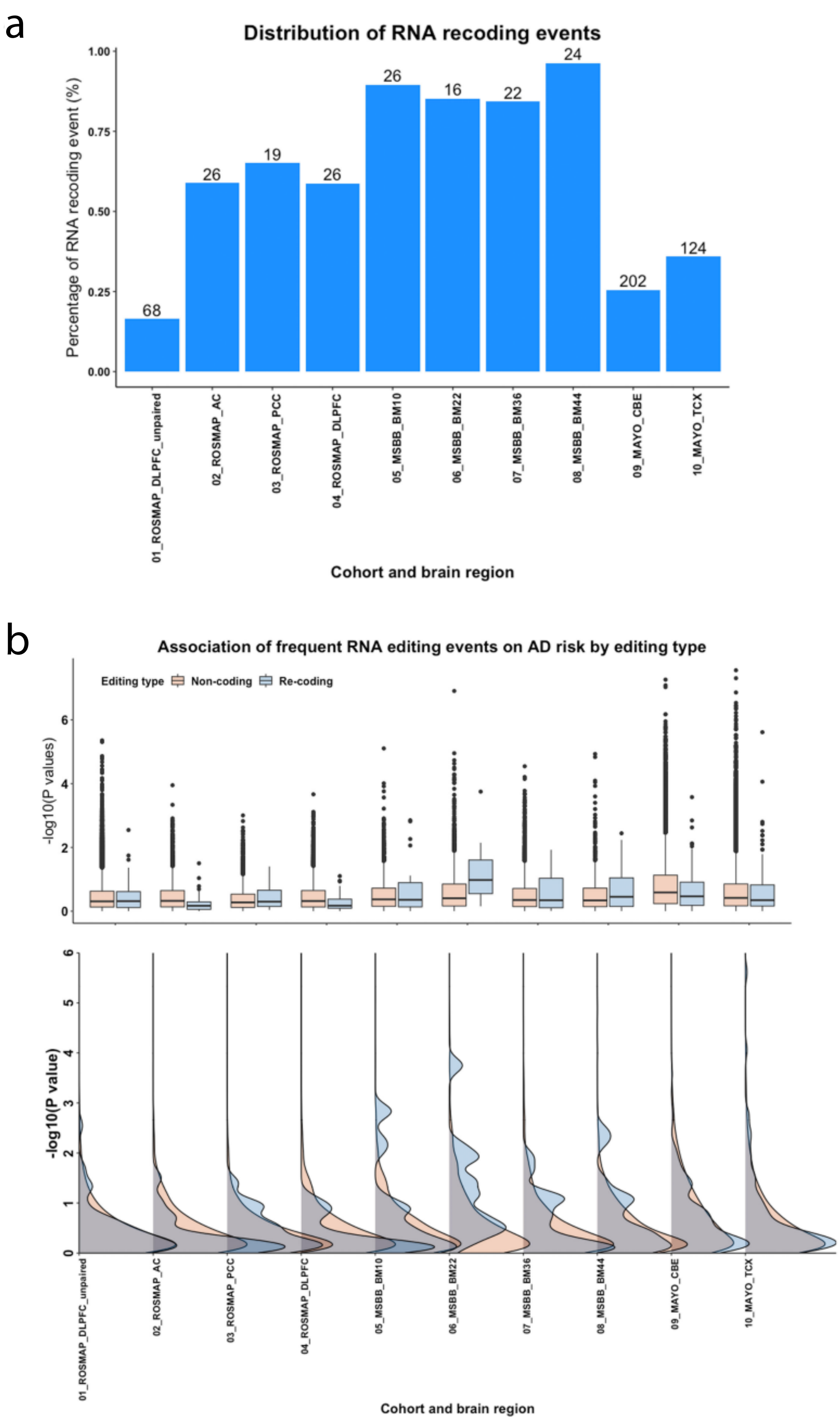
a
Distribution of RNA recoding events
Percentage of RNA recoding event (%)
1.00
0.75
0.50
0.25
0.00
68
26
19
26
26
16
22
24
202
124
01_ROSMAP_DLPFC_unpaired
02_ROSMAP_AC
03_ROSMAP_PCC
04_ROSMAP_DLPFC
05_MSBB_BM10
06_MSBB_BM22
07_MSBB_BM36
08_MSBB_BM44
09_MAYO_CBE
10_MAYO_TCX
Cohort and brain region
b
Association of frequent RNA editing events on AD risk by editing type
Editing type
Non-coding
Re-coding
-log10(P values)
-log10(P value)
01_ROSMAP_DLPFC_unpaired
02_ROSMAP_AC
03_ROSMAP_PCC
04_ROSMAP_DLPFC
05_MSBB_BM10
06_MSBB_BM22
07_MSBB_BM36
08_MSBB_BM44
09_MAYO_CBE
10_MAYO_TCX
Cohort and brain region

Fig. S7. Functions of the principal components derived from the top AD associated editing events on the expressions of genes, isoforms and proteins. The matrix plot shows the associations of the 7 principal components (PCs) derived from the 7 top RNA editing events with expressions of the mRNA and protein expressions of the genes (a,c) and isoforms (b) harboring the editing event. The signed –log10(*P*) values were presented and coded for different colors where white was for values between -1.3 to 1.3 (*P*=0.05), darkening blue was for negative values from 0, and darkening red was for positive values from 0. The displayed *P* values were not adjusted by the multiple testings and were derived by two-sided tests.

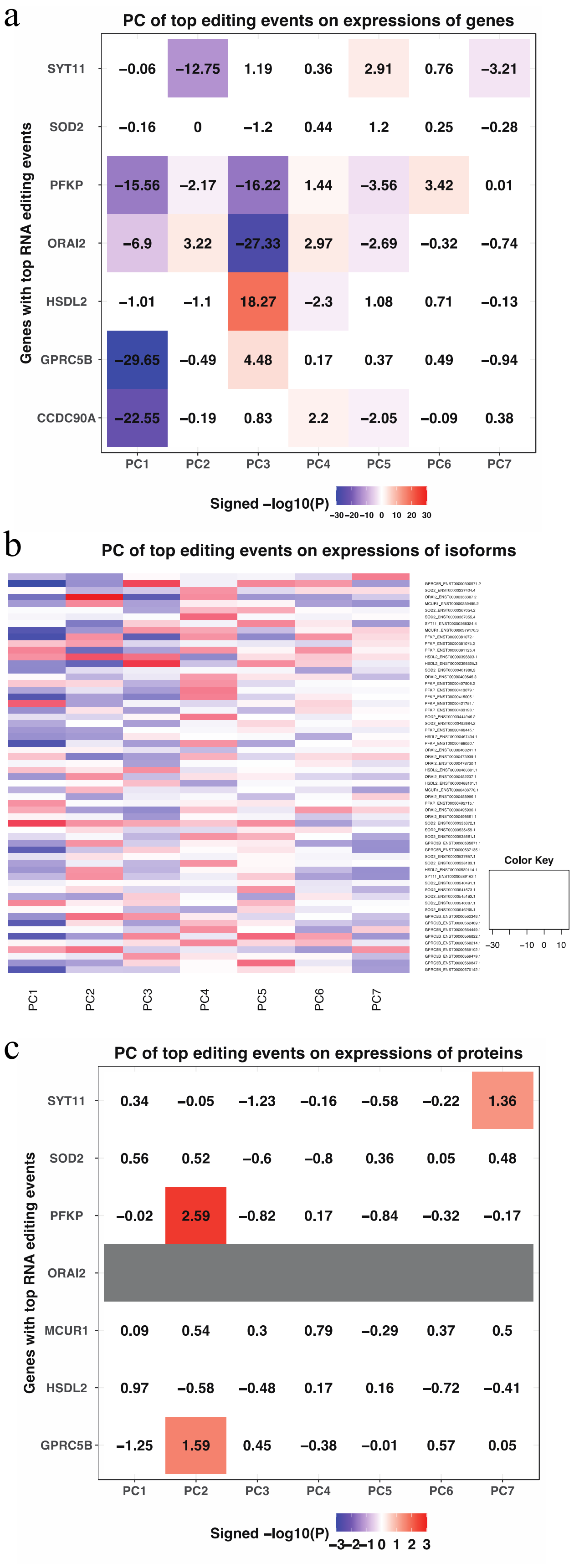
a
PC of top editing events on expressions of genes
Genes with top RNA editing events
SYT11 −0.06 −12.75 1.19 0.36 2.91 0.76 −3.21
SOD2 −0.16 0 −1.2 0.44 1.2 0.25 −0.28
PFKP −15.56 −2.17 −16.22 1.44 −3.56 3.42 0.01
ORAI2 −6.9 3.22 −27.33 2.97 −2.69 −0.32 −0.74
HSDL2 −1.01 −1.1 18.27 −2.3 1.08 0.71 −0.13
GPRC5B −29.65 −0.49 4.48 0.17 0.37 0.49 −0.94
CCDC90A −22.55 −0.19 0.83 2.2 −2.05 −0.09 0.38
PC1 PC2 PC3 PC4 PC5 PC6 PC7
Signed −log10(P)
−30 −20 −10 0 10 20 30
b
PC of top editing events on expressions of isoforms
Color Key
−30 −10 0 10
PC1 PC2 PC3 PC4 PC5 PC6 PC7
c
PC of top editing events on expressions of proteins
Genes with top RNA editing events
SYT11 0.34 −0.05 −1.23 −0.16 −0.58 −0.22 1.36
SOD2 0.56 0.52 −0.6 −0.8 0.36 0.05 0.48
PFKP −0.02 2.59 −0.82 0.17 −0.84 −0.32 −0.17
ORAI2
MCUR1 0.09 0.54 0.3 0.79 −0.29 0.37 0.5
HSDL2 0.97 −0.58 −0.48 0.17 0.16 −0.72 −0.41
GPRC5B −1.25 1.59 0.45 −0.38 −0.01 0.57 0.05
PC1 PC2 PC3 PC4 PC5 PC6 PC7
Signed −log10(P)
−3 −2 −1 0 1 2 3

111

Fig. S8. Mass-spectrometry figure of edited peptide (frequency ≥50%) hits in ROSMAP TMT proteomic dataset. The edited peptide hits are mapped to RNA editing events at *CADPS* (chr3:62860434, upper left), *GRIA3* (chrX:122598962, upper right), *CACNG8* (chr19:54485579, middle left), *GRIA3* (chrX:122598962, middle right), *GRIA2* (chr4:158281294, bottom left and right). Within each mass-spectrometry plot, X axis represents the ratio of mass to charge number of y and b ions, and the Y axis represent the relative abundance of each peak.

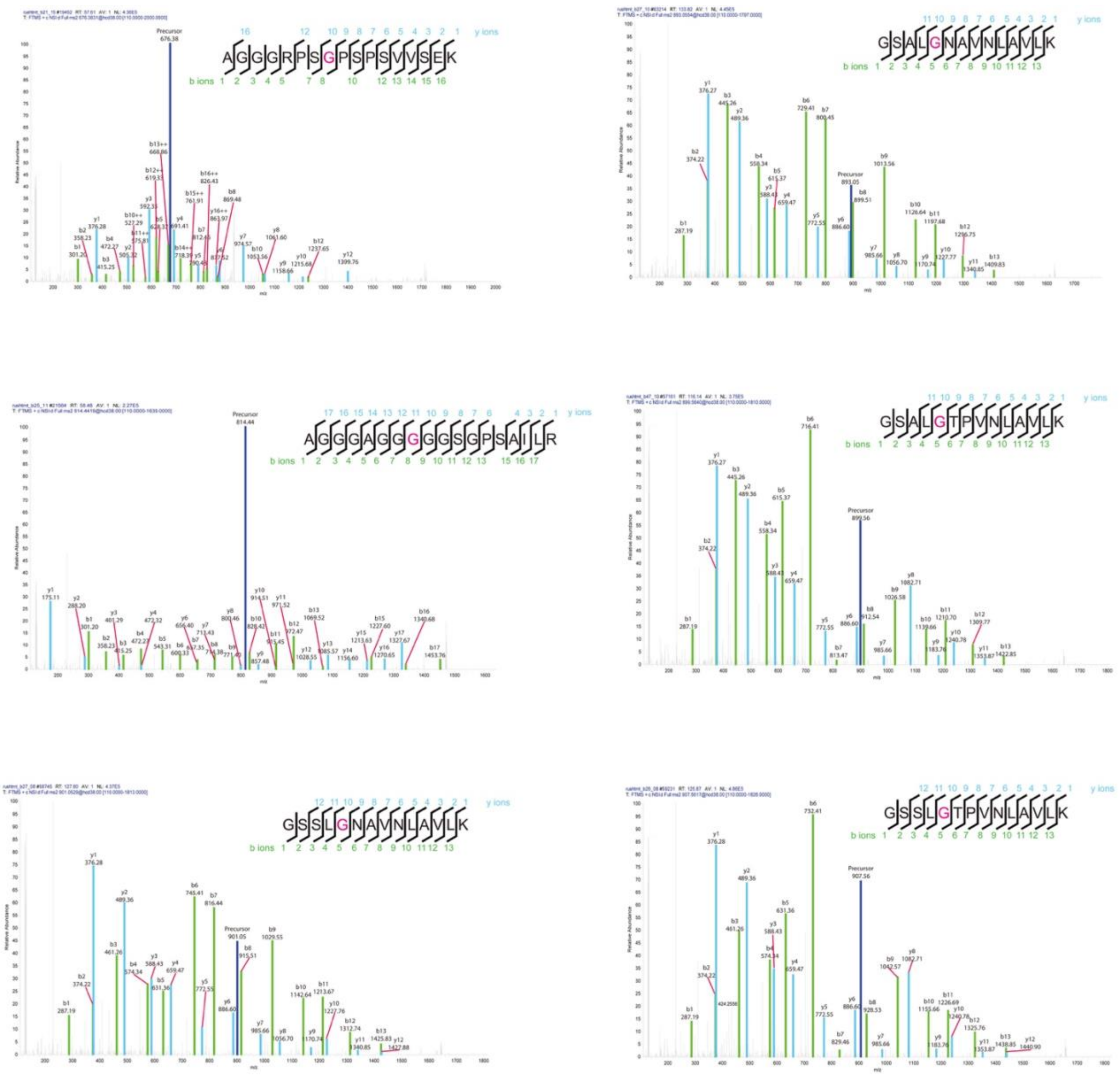

Table S1. Characteristic of the cohorts and picard metrics of RNA-seq datasets.

Characteristics of cohorts

Data type	Study	Brain region	AD status	N	Age at death (years)*	Female (N, %)*
Paired	MAYO	CBE	All	229	79.62 (8.23)	112, 48.91
		TCX	Normal controls	62	82.55 (8.30)	29, 46.77
			MCI	0	NA	NA
			AD cases	65	82.29 (7.16)	38, 58.46
			PSP	102	76.14 (7.52)	45, 44.12
	MSBB	BM10	All	142	83.05 (7.85)	93, 65.49
		BM22	Normal controls	20	82.45 (8.15)	12, 60
		BM36	MCI	24	79.29 (10.53)	11, 45.83
		BM44	AD cases	98	84.1 (6.76)	70, 71.43
	ROSMAP	AC	All	68	91.35 (6.78)	42, 61.76
		DLPFC	Normal controls	23	90.67 (6.55)	11, 47.83
		PCC	MCI	15	95.05 (4.63)	11, 73.33
			AD cases	29	89.81 (7.39)	19, 65.52
Unpaired	ROSMAP	DLPFC	All	635	88.71 (6.65)	405, 63.78
			Normal controls	200	86.11 (6.75)	122, 61
			MCI	167	88.84 (6.04)	105, 62.87
			AD cases	255	90.73 (6.26)	172, 67.45

Picard metrics of RNA-seq datasets

Picard metrics / Data type	Study	Brain region	Mean (SD)#	Comparison to MAYO CBE		
				BETA#	SE#	*P*#
**Total reads (N)**						
Paired	MAYO	CBE	45762142(8917944)	reference	reference	reference
		TCX	45002272(8941394)	-759869.8	732325	3.00E-01
	MSBB	BM10	17530795(5048660)	-28231347.4	837011.9	6.72E-195
		BM22	16583964(4761792)	-29178177.8	837011.9	3.23E-205
		BM36	15810183(4292940)	-29951959.3	837011.9	1.06E-213
		BM44	21690336(7302082)	-24071806.6	837011.9	1.01E-150
	ROSMAP	AC	36312847(5129869)	-9449295.4	1082212.7	5.50E-18
		DLPFC	39105695(10822802)	-6656447.6	1082212.7	9.42E-10
		PCC	37943854(5685988)	-7818288.2	1082212.7	7.32E-13
Unpaired	ROSMAP	DLPFC	28278275(8778985)	-17483867.5	604030.5	2.46E-152
**Aligned reads (N)**						
Paired	MAYO	CBE	45185927(8770988)	reference	reference	reference

		TCX	44501837(8813148)	-684090.2	717854.6	3.41E-01
	MSBB	BM10	17314608(5038827)	-27871318.7	820473	4.31E-197
		BM22	16412370(4752375)	-28773557.2	820473	3.88E-207
		BM36	15656050(4260491)	-29529876.6	820473	1.31E-215
		BM44	21518386(7297171)	-23667541.2	820473	1.78E-151
	ROSMAP	AC	34444757(5010850)	-10741170	1060828.7	1.72E-23
		DLPFC	37204812(10833602)	-7981115.3	1060828.7	8.27E-14
		PCC	35986334(5548391)	-9199592.6	1060828.7	9.09E-18
Unpaired	ROSMAP	DLPFC	27160757(8495879)	-18025170	592095.1	1.93E-165
Uniquely aligned reads (N)						
Paired	MAYO	CBE	41769995(8130814)	reference	reference	reference
		TCX	40915115(8147291)	-854880.4	661521.3	1.96E-01
	MSBB	BM10	14178561(4104213)	-27591434.6	756086.7	3.14E-220
		BM22	13279733(4045273)	-28490262.6	756086.7	3.13E-231
		BM36	12834868(3397540)	-28935126.9	756086.7	1.08E-236
		BM44	16667204(5682457)	-25102791.3	756086.7	4.12E-190
	ROSMAP	AC	32777825(4724429)	-8992169.8	977580.6	9.48E-20
		DLPFC	35330631(10327461)	-6439364.4	977580.6	5.83E-11
		PCC	34154475(5230901)	-7615519.8	977580.6	1.11E-14
Unpaired	ROSMAP	DLPFC	25841144(8081045)	-15928851	545630.7	1.72E-154
Ribosomal bases (%)						
Paired	MAYO	CBE	1.79(0.64)	reference	reference	reference
		TCX	2.65(0.97)	0.8643061	0.1707043	4.53E-07
	MSBB	BM10	0.74(3.44)	-1.0519114	0.1951067	7.88E-08
		BM22	0.83(1.54)	-0.9601917	0.1951067	9.35E-07
		BM36	0.37(0.46)	-1.4234333	0.1951067	4.38E-13
		BM44	1.27(4.76)	-0.5242833	0.1951067	7.27E-03
	ROSMAP	AC	0.6(0.18)	-1.1944694	0.2522628	2.36E-06
		DLPFC	0.7(0.21)	-1.0902018	0.2522628	1.63E-05
		PCC	0.58(0.24)	-1.2066312	0.2522628	1.86E-06
Unpaired	ROSMAP	DLPFC	2.89(1)	1.1036106	0.140799	7.64E-15
Median 3' bias						
Paired	MAYO	CBE	0.68(0.07)	reference	reference	reference
		TCX	0.7(0.07)	0.01959141	0.01411924	1.65E-01
	MSBB	BM10	0.96(0.07)	0.27558379	0.01613761	7.66E-61
		BM22	0.94(0.07)	0.25440768	0.01613761	1.21E-52
		BM36	0.93(0.05)	0.24507238	0.01613761	3.48E-49
		BM44	0.86(0.07)	0.18117453	0.01613761	2.41E-28
	ROSMAP	AC	0.56(0.04)	-0.12711676	0.02086508	1.35E-09

		DLPFC	0.63(0.05)	-0.05676807	0.02086508	6.57E-03
		PCC	0.61(0.05)	-0.07113098	0.02086508	6.66E-04
Unpaired	ROSMAP	DLPFC	1.3(0.24)	0.61886072	0.01164572	0.00E+00

Correlation between number of editing events and total reads

Data type	Study	Brain region	N of subjects	N of editing events (mean,SD)	*R*	*P*
Paired	MAYO	CBE	229	27060.24, 10538.6	0.5	7.57E-16
		TCX	229	12490.45, 3330.1	0.69	< 2.2E-16
	MSBB	BM10	142	857.95, 397.52	0.78	< 2.2E-16
		BM22	142	602.19, 198.07	0.78	< 2.2E-16
		BM36	142	771.55, 302.95	0.78	< 2.2E-16
		BM44	142	872.48, 344.30	0.86	< 2.2E-16
	ROSMAP	AC	68	1544.57, 613.03	0.16	0.19
		DLPFC	68	1474.37, 675.52	0.29	0.01
		PCC	68	881.37, 446.84	0.02	0.86
Unpaired	ROSMAP	DLPFC	635	12257.07, 7006.66	0.31	3.00E-15

*Age at death is represented by mean (SD) and female is represented by number (N) and percentage (%)
#BETA represent the difference in each picard metric between MAYO CBE dataset compared to all the other 9 datasets, and the SE and P represent the corresponding standard error and *P* value. The displayed *P* values were not adjusted by the multiple testings and were derived by two-sided tests.
Abbreviation: SD, standard deviation; MAYO, Mayo RNAseq study; CBE, cerebellum; TCX, temporal cortex; MSBB, Mount Sinai Brain Bank; AC, anterior cingulate cortex; DLPFC, dorsolateral prefrontal cortex; PCC, posterior cingulate cortex; PSP, progressive supranuclear palsy.

Table S2. The recoding RNA editing events with proteomic peptide hits.

Annotation^					
Unique identifier	NM	CDS	Exon number	DNA substitute	Amino acid substitute
chr1_16133978_UQCRHL	NM_001089591.1	51 to 326	1	A65G	E22G
chr1_110256304_GSTM5_peptide1	NM_000851.3	72 to728	5	A281G	K94R
chr1_110256304_GSTM5_peptide2					
chr1_160302244_COPA	NM_001098398.1	395 to 4096	6	A490G	I164V
chr3_62423807_CADPS	NM_183393.2	361 to 4185	25	A3512G	E1171G
chr3_62860434_CADPS	NM_183393.2	361 to 4185	1	A271G	S91G
chr4_158281294_GRIA2_peptide1	NM_001083619.1	460 to 3111	13	A2290G	R764G
chr4_158281294_GRIA2_peptide2	NM_000826.3 / NM_001083620.1	460 to 3111 / 112 to 2622	13	A2290G / A2149G	R764G / R717G
chr6_26104432_HIST1H4C	NM_003542.3	1 to 312	1	A257G	D86G
chr19_47152737_DACT3	NM_001301046.1	442 to 1656	4	A217G	S73G
chr19_54485579_CACNG8	NM_031895.5	108 to 1385	4	A754G	S252G
chrX_122598962_GRIA3_peptide1	NM_000828.4	294 to 2978	13	A2323G	R775G

chrX_122598962_GRIA3_peptide2	NM_007325.4	294 to 2978	13	A2323G	R775G

Annotation^

Unique identifier	RNA sequence[&]	Edited peptide sequence	Non-edited peptide sequence
chr1_16133978_UQCRHL	75atgcttaccgaatccggagatcctgaggaggaggaagaggA/[G]agaggaggaattagtggatcccctaacaacagtgagag253	9MLTESGDPEEEEEGEEELVDPLTTVR24	9MLTESGDPEEEEEEEEELVDPLTTVR24
chr1_110256304_GSTM5_peptide1	316caagcacaacctgtgtggggagacagaagaggagaA/[G]ga354	83KHNLCGETEEER94	83KHNLCGETEEEK94
chr1_110256304_GSTM5_peptide2		84HNLCGETEEER94	84HNLCGETEEEK94
chr1_160302244_COPA	875gtttgggatA/[G]tttctggtctgagg898	161VWDVSGLR168	161VWDISGLR168
chr3_62423807_CADPS	3863aggtcaatgA/[G]ggagatgtacatagaaaggt3892	1169VNGEMYIER1177	1169VNEEMYIER1177
chr3_62860434_CADPS	610gctggcggcggccggccctccA/[G]gccccagcccgtcggtggtgagcgagaag660	84AGGGRPSGPSPSVVSEK100	84AGGGRPSSPSPSVVSEK100
chr4_158281294_GRIA2_peptide1	2737ggatcctcattaaA[/G]aaatgcggttaacctcgcagtactaaaa2778	760GSSLGNAVNLAVLK773	765NAVNLAVLK773
chr4_158281294_GRIA2_peptide2	2737/2248ggatcctcattaaA[/G]aaccccagtaaatcttgcagtattgaaa2778/2289	760/713GSSLGTPVNLAVLK773/726	765/718NAVNLAVLK773/726
chr6_26104432_HIST1H4C	240aactgtcacagccatggA/[G]tgtagtatatgccctaaaac277	81TVTAMGVVYALK92	81TVTAMDVVYALK92
chr19_47152737_DACT3	625cagcggccgcccgacgcgtctccgtcccccggcA/[G]gcgcgcgacccgcgcgg675	62QRPPDASPSPGGARPAR78	62QRPPDASPSPGSARPAR78
chr19_54485579_CACNG8	840gccggcgggggcgcgggcggcA/[G]gtggcgggagcggcccctcggccatcctccgt893	245AGGGAGGGGGSGPSAILR262	245AGGGAGGSGGSGPSAILR262
chrX_122598962_GRIA3_peptide1	2604ggctcagcattagA/[G]aaatgctgttaacctggcagtattaaaa2645	771GSALGNAVNLAVLK784	776NAVNLAVLK784
chrX_122598962_GRIA3_peptide2	2604ggctcagcattagA/[G]aacgcctgtaaaccttgcagtattgaaa2645		776TPVNLAVLK784

Concordance, correlation, and expression levels

Unique identifier	Concordance$				Correlation*		Expression level#		
	N1, %	N2,%	N3, %	N4, %	r	P	Edited RNA reads (%)	Edited peptide quantity (abundance)	Non_edited peptide quantity (abundance)
chr1_16133978_UQCRHL	13 , 7.6 %	84 , 49.12 %	60 , 35.09 %	14 , 8.19 %	0.07	0.39	33.67±40.05	13.5±2.73	7.7±1.87
chr1_110256304_GSTM5_peptide1	0 , 0 %	164 , 95.91 %	5 , 2.92 %	2 , 1.17 %	0.02	0.83	0.46±2.34	56.48±37.39	95.97±75.13
chr1_110256304_GSTM5_peptide2	2 , 1.17 %	89 , 52.05 %	3 , 1.75 %	77 , 45.03 %	0.03	0.70		44.98±14.25	585.09±446.40

chr1_160302244_COPA	23 , 13.45 %	54 , 31.58 %	74 , 43.27 %	20 , 11.7 %	0.08	0.30	13.27±13.80	127.3±8.2	239.06±20.12
chr3_62423807_CADPS	47 , 27.49 %	30 , 17.54 %	82 , 47.95 %	12 , 7.02 %	-0.05	0.49	14.67±9.95	177.36±17.56	507.38±45.40
chr3_62860434_CADPS	0 , 0 %	19 , 11.11 %	0 , 0 %	152 , 88.89 %	NA	NA	0.03±0.73	229.97±23.26	929.77±49.22
chr4_158281294_GRIA2_peptide1	114 , 66.67 %	2 , 1.17 %	15 , 8.77 %	40 , 23.39 %	-0.09	0.26	0.30±0.20	738.08±62.41	581.38±48.07
chr4_158281294_GRIA2_peptide2	114 , 66.67 %	2 , 1.17 %	15 , 8.77 %	40 , 23.39 %	-0.08	0.29		500.35±69.07	197.64±20.95
chr6_26104432_HIST1H4C	1 , 0.58 %	167 , 97.66 %	0 , 0 %	3 , 1.75 %	-0.29	9.68E-05	0.07±1.83	54.57±52.73	2003.41±916.83
chr19_47152737_DACT3	2 , 1.17 %	157 , 91.81 %	5 , 2.92 %	7 , 4.09 %	0.24	1.40E-03	3.82±15.58	18.04±4.62	NA
chr19_54485579_CACNG8	18 , 10.53 %	15 , 8.77 %	2 , 1.17 %	136 , 79.53 %	0.02	0.78	2.15±6.33	85.89±11.66	501.95±48.83
chrX_122598962_GRIA3_peptide1	83 , 48.54 %	6 , 3.51 %	11 , 6.43 %	71 , 41.52 %	0.21	6.74E-03	49.78±45.77	577.77±44.71	581.38±48.07
chrX_122598962_GRIA3_peptide2	83 , 48.54 %	6 , 3.51 %	11 , 6.43 %	71 , 41.52 %	-0.07	0.36		545.44±64.34	197.64±20.95

AD association@

Unique identifier	Reported event	Reported AD association	Peptide			RNA			
			BETA	STDERR	P	N	BETA	STDERR	P
chr1_16133978_UQCRHL	No	No	-0.007	0.03	0.84	269	-2.22	1.71	0.20
chr1_110256304_GSTM5_peptide1	Yes	No	0.004	0.01	0.66	25	0.32	0.12	6.40E-03
chr1_110256304_GSTM5_peptide2			0.492	0.20	0.01				
chr1_160302244_COPA	Yes	Yes	-0.047	0.04	0.22	346	1.31	0.65	0.04
chr3_62423807_CADPS	Yes	No	0.000	0.07	0.995	469	-0.85	0.43	0.05
chr3_62860434_CADPS	Yes	No	0.024	0.07	0.72	1	-0.05	0.04	0.22
chr4_158281294_GRIA2_peptide1	Yes	Yes	-0.014	0.02	0.46	458	-1.15	0.85	0.18
chr4_158281294_GRIA2_peptide2			-0.022	0.05	0.67				
chr6_26104432_HIST1H4C	No	No	-0.093	0.10	0.33	1	-0.07	0.09	0.47
chr19_47152737_DACT3	Yes	No	-0.156	0.09	0.09	38	-0.18	0.75	0.81
chr19_54485579_CACNG8	Yes	No	-0.010	0.08	0.91	72	-0.04	0.30	0.89
chrX_122598962_GRIA3_peptide1	Yes	Yes	-0.022	0.01	0.04	346	-5.40	2.24	0.02

chrX_122598962_GRIA3_peptide2	-0.028	0.06	0.62

^On both the RNA and peptide level, the nucleotide and amino acid at the editing site were represented by the two red characters separated by slash. The left character is the non-edited one while the right character in the bracket is the edited one.
$N1, %: Number and percentage of subjects with detection of editings on both RNA and peptide level; N2, %: Number and percentage of subjects without detection of editings on either RNA or peptide level; N3, %: Number and percentage of subjects with detection of editing on RNA but not peptide level; N4, %: Number and percentage of subjects with detection of editing on peptide but not RNA level. Chi-square and corresponding P values for the contingency table of the four numbers are displayed.
*r and P represent the correlation coefficient and corresponding P value between fraction of edited RNA read and abundance.
#Values are represented by mean ± SD. Edited RNA reads were represented by the percentage of alternative read over the total amount of reads at that locus. On the peptide level, unit are shown in both the normalized abundance.
@N represent the number of subjects (out of total 635) carrying the editing event. BETA, STDERR, and P represent the regression coefficient, standard error and corresponding P values of the generalized linear model of RNA editing level (log2 of the ratio of the normalized abundance of the edited peptide over the nonedited peptide and % alternative allele on RNA level) as the dependent variable and clinical AD status (0, 1, and 2 represent normal controls, mild cognitive impairment (MCI), and AD patients) as exposure variable, and covariates include sex, age at death, postmortem interval (PMI), study (ROS or MAP), RIN and experimental batch (for RNA dataset). Peptide analysis is within 166 subjects with available data of all the variables included in the analysis and also those subjects with RNA-seq datasets. RNA-seq analysis include 620 subjects with RNA-seq datasets and all the included variables. All the statistical tests are two-sided.

Table S3. Regional differences in gene expressions of *ADARs*.

Study	Comparison	ADAR genes	N	BETA[&]	STDERR[&]	*P*[&]
ROSMAP multi-region[#] (N=68)	AC - DLPFC	*ADAR1*	68	-0.64	1.21	0.6
		ADAR2	68	-2.16	0.32	1.20E-11
		ADAR3	68	0.25	0.36	0.49
		ADARs[$]	68	-3.01	1.44	0.04
	PCC - DLPFC	*ADAR1*	68	-8.14	1.06	1.80E-14
		ADAR2	68	-2.24	0.3	6.20E-14
		ADAR3	68	-2.25	0.32	1.00E-12
		ADARs[$]	68	-8.11	1.34	1.40E-09
	PCC - AC	*ADAR1*	68	-7.07	1.18	1.90E-09
		ADAR2	68	-0.07	0.28	0.79
		ADAR3	68	-2.45	0.36	1.30E-11
		ADARs[$]	68	-4.68	1.41	9.30E-04
MSBB* (N=142)	BM36 - BM44	*ADAR1*	142	-966	252	1.20E-04
		ADAR2	142	-160	49.7	1.30E-03
		ADAR3	142	-341	57.7	3.60E-09
		ADARs[$]	142	-778	264	3.30E-03
	BM36 - BM10	*ADAR1*	142	-146	193	0.45
		ADAR2	142	-148	35.8	3.90E-05
		ADAR3	142	104	37.1	4.90E-03
		ADARs[$]	142	-396	198	0.05
	BM36 - BM22	*ADAR1*	142	121	142	0.39
		ADAR2	142	-132	31.6	3.00E-05
		ADAR3	142	26.3	30.8	0.39
		ADARs[$]	142	-41.5	150	0.78
	BM22 - BM10	*ADAR1*	142	-238	183	0.19
		ADAR2	142	-17.8	37.9	0.64
		ADAR3	142	93.7	32.1	3.50E-03
		ADARs[$]	142	-350	195	0.07
	BM44 - BM10	*ADAR1*	142	937	254	2.20E-04
		ADAR2	142	17.9	49.3	0.72
		ADAR3	142	457	54.9	1.10E-16
		ADARs[$]	142	491	260	0.06
	BM22 - BM44	*ADAR1*	142	-1030	231	9.10E-06
		ADAR2	142	-32.1	51.2	0.53
		ADAR3	142	-318	50.2	2.40E-10

		ADARs$	142	-767	244	1.70E-03
MAYO* (N=229)	TCX - CBE	*ADAR1*	229	-2250	325	3.90E-12
		ADAR2	229	-7500	263	<2.2E-16
		ADAR3	229	1460	145	<2.2E-16
		ADARs$	229	-11200	578	<2.2E-16

#Gene expression units for ROSMAP multi-region study are represented by transcripts per million (TPM) values obtained through RSEM tool with the reference transcriptome of GENCODE v24.

*Gene expression units for MSSB and MAYO RNA-seq study are represented by counts of the reads of the gene using STAR tool by setting quantMode as GeneCounts according to the reference transcriptome of GENCODE v24.

$ ADARs = ADAR1+ADAR2-ADAR3.

& The BETA, STDERR, and *P* represent the regression coefficient, standard error, and the corresponding P values for the exposure variable of region (coded as 1 for one brain region and 0 for the other brain region) in the general linear mixed model using R lme package with dependent variable of gene expression levels of ADARs, random effect of subject ID, and fixed effects of the covariates including age at death, sex, postmortem interval (PMI), and RIN score. The displayed *P* values were not adjusted by the multiple testings and were derived by two-sided tests.

Table S4. Top RNA editing events associated with AD status.

Associations with AD status in Stage I and II[#]

Gene: top RNA editing	Outcome	Stage I			Stage II			
		N	BETA (STDERR)	*P*	N	BETA (STDERR)	Direction	*P*
SYT11:chr1_155851645	AD status in all	620	1.36 (0.32)	2.09E-05	924	1.66 (0.26)	"++++"	2.94E-10
MCUR1:chr6_13788361	AD status in all	620	2.30 (0.60)	1.36E-04	924	2.75 (0.46)	"++++"	1.66E-09
SOD2:chr6_13788361	AD status in all	620	1.31 (0.31)	2.75E-05	924	1.80 (0.27)	"++++"	1.96E-11
ORAI2:chr7_102096952	AD status in all	620	2.94 (0.64)	4.64E-06	782	2.74 (0.55)	"+x++"	4.80E-07
HSDL2:chr9_115237504	AD status in all	620	5.13 (1.33)	1.25E-04	782	5.23 (1.04)	"+x++"	5.49E-07
PFKP:chr10_3168677	AD status in all	620	4.89 (1.44)	7.54E-04	924	5.59 (1.05)	"++++"	1.08E-07
SYT11:chr1_155851645	AD status in females	398	1.98 (0.41)	1.65E-06	581	2.23 (0.34)	"++++"	3.60E-11
MCUR1:chr6_13788361	AD status in females	398	2.74 (0.78)	5.22E-04	581	2.98 (0.57)	"++++"	2.10E-07
SOD2:chr6_13788361	AD status in females	398	1.81 (0.39)	4.33E-06	581	2.13 (0.34)	"++++"	3.08E-10
GPRC5B:chr16_19876365	AD status in females	398	5.59 (1.17)	2.68E-06	448	5.87 (1.14)	"+x+x"	2.79E-07

Functional associations in STAGE I unpaired ROSMAP DLPFC samples*

Gene: top RNA editing	Outcome Category	Outcome	N	BETA	STDERR	*P*
SYT11:chr1_155851645	ADARs gene expression	*ADAR1*	505	0.0427	0.0164	0.00938
		ADAR2	505	-0.169	0.0197	1.14E-16
		ADAR3	505	0.308	0.0538	1.75E-08
	Target gene expression	*SYT11*	505	0.166	0.0334	9.60E-07
	Target isoform expression	*ENST00000368324.4*	526	4.37	1.46	0.00284
	Target protein expression	SYT11_Q9BT88	78	-0.06	0.02	0.01
SOD2:chr6_13788361	ADARs gene expression	*ADAR1*	505	0.00502	0.0175	0.774
		ADAR2	505	-0.0824	0.0221	0.00021
		ADAR3	505	0.19	0.0582	0.00119
	Target gene expression	*SOD2*	505	0.0517	0.0493	0.295
	Target isoform expression	*ENST00000546260.1*	526	-0.0528	0.0252	0.0369
	Target protein expression	SOD2_P04179	78	-0.02	0.03	0.54
PFKP:chr10_3168677	ADARs gene expression	*ADAR1*	505	0.0481	0.0164	0.00346
		ADAR2	505	0.0158	0.0211	0.455

		ADAR3	505	0.0959	0.0554	0.0841
	Target gene expression	*PFKP*	505	0.388	0.0376	9.17E-23
	Target isoform expression	*ENST00000381072.1*	526	22.3	1.97	1.00E-26
	Target protein expression	PFKP_Q01813	78	-0.01	0.02	0.61
ORAI2:chr7_102096952		*ADAR1*	505	-0.0412	0.0175	0.0188
	ADARs gene expression	*ADAR2*	505	0.0101	0.0225	0.654
		ADAR3	505	0.146	0.0588	0.0132
	Target gene expression	*ORAI2*	505	0.254	0.0334	1.43E-13
	Target isoform expression	*ENST00000356387.2*	526	2.43	0.221	2.04E-25
	Target protein expression	Protein	78	NA	NA	NA
MCUR1:chr6_13788361		*ADAR1*	505	0.0568	0.0163	0.000555
	ADARs gene expression	*ADAR2*	505	-0.0426	0.0211	0.0438
		ADAR3	505	0.11	0.0554	0.0468
	Target gene expression	*MCUR1*	505	0.0663	0.0197	0.000823
	Target isoform expression	*ENST00000379170.3*	526	1.22	0.216	2.40E-08
	Target protein expression	MCUR1_Q96AQ8	78	-0.08	0.04	0.05
HSDL2:chr9_115237504		*ADAR1*	505	-0.043	0.0189	0.0233
	ADARs gene expression	*ADAR2*	505	-0.126	0.0237	1.67E-07
		ADAR3	505	0.175	0.0635	0.00594
	Target gene expression	*HSDL2*	505	0.201	0.0367	6.81E-08
	Target isoform expression	*ENST00000398803.1*	526	1.25	0.305	4.93E-05
	Target protein expression	HSDL2_Q6YN16	78	-0.04	0.03	0.13
GPRC5B:chr16_19876365		*ADAR1*	505	-0.0318	0.0167	0.0573
	ADARs gene expression	*ADAR2*	505	-0.0568	0.0213	0.00792
		ADAR3	505	0.233	0.0555	3.26E-05
	Target gene expression	*GPRC5B*	505	0.155	0.0438	0.000422
	Target isoform expression	*ENST00000300571.2*	526	17.8	3.62	1.16E-06
	Target protein expression	GPRC5B.Q9NZH0.2	78	0.16	0.08	0.05

Associations in STAGE I unpaired ROSMAP DLPFC samples before and after adjustment of the neuron proportion or total reads

Gene: top RNA editing	Outcome	Unadjusted *P*	Adjusted neuron transcription module			Adjusted total reads		
			N	BETA (STDERR)	P	N	BETA (STDERR)	*P*

SYT11:chr1_155851645	AD status in all#	2.09E-05	493	1.21 (0.36)	7.05E-04	614	1.34 (0.32)	3.27E-05
MCUR1:chr6_13788361		1.36E-04	493	2.29 (0.69)	9.99E-04	614	2.21 (0.60)	2.32E-04
SOD2:chr6_13788361		2.75E-05	493	1.12 (0.35)	1.24E-03	614	1.31 (0.31)	3.30E-05
ORAI2:chr7_102096952		4.64E-06	493	2.49 (0.71)	4.90E-04	614	3.02 (0.63)	2.07E-06
HSDL2:chr9_115237504		1.25E-04	493	4.81 (1.49)	1.38E-03	614	0.52 (0.01)	1.01E-04
PFKP:chr10_3168677		7.54E-04	493	4.39 (1.65)	8.04E-03	614	0.48 (0.01)	9.38E-04
GPRC5B:chr16_19876365	AD status in females#	2.68E-06	306	5.45 (1.42)	1.48E-04	392	0.57 (0.01)	2.40E-06
ORAI2:chr7_102096952	PHFtau in all*	4.72E-08	500	0.11 (0.03)	3.66E-05	622	0.13 (0.02)	8.07E-08
KCNIP2:chr10_103596067		6.72E-07	500	0.06 (0.01)	1.10E-05	622	0.07 (0.01)	1.08E-06
GPRC5B:chr16_19874115		5.54E-07	500	0.12 (0.03)	1.14E-04	622	0.15 (0.03)	6.59E-07
YPEL1:chr22_22078228		1.77E-07	500	0.17 (0.03)	1.03E-06	622	0.18 (0.03)	9.39E-08
AC174470.1:chr17_79780692	β-amyloid in all*	9.38E-07	500	-0.08 (0.02)	2.23E-04	622	-0.09 (0.02)	8.51E-07
ORAI2:chr7_102096952	Neuritic plaque burden in all*	8.47E-07	505	0.008 (0.002)	1.59E-05	628	8.37E-03 (1.61E-04)	2.97E-07
CABP1:chr12_121078907		2.74E-07	505	-0.009 (0.002)	2.08E-05	628	-9.34E-03 (1.84E-04)	5.41E-07
AC174470.1:chr17_79780692	Cognition decline*	5.76E-07	481	0.002 (6E-04)	1.82E-03	593	2.35E-03 (4.7E-04)	7.44E-07
MUM1:chr19_1371887		8.50E-07	481	-7E-04 (1E-04)	9.95E-06	593	-6.88E-04 (1.37E-04)	6.65E-07

#Top RNA editing events associated with AD risk in Stage I and II genome-wide analysis in all subjects and female only (Stage II meta-P ≤ 1.2×10-6). Stage I dataset include ROSMAP unpaired samples of dorsolateral prefrontal cortex (DLPFC), and the Stage II datasets include samples from Stage I and BM44 of MSBB study (MSBB_BM44), temporal cortex (TCX) of MAYO study (MAYO_TCX), and DLPFC samples of independent set of subjects participating in the ROSMAP multi-region study (ROSMAP multi-region DLPFC). BETA, STDERR, and P represent the regression coefficient, standard error and corresponding P values of the generalized linear model of RNA editing level (% alternative read) as the dependent variable and clinical AD status (0, 1, and 2 represent normal controls, mild cognitive impairment (MCI), and AD patients) as exposure variable, and covariates include sex, age at death, postmortem interval (PMI), RIN, experimental batch, study (in 2 of the ROSMAP DLPFC datasets), race (in MSBB BM44 dataset), and tissue source (in MAYO temporal cortex dataset). The direction column in Stage II showed the sign of the BETA from each included dataset in the order of ROSMAP unpaired DLPFC, MSBB_BM44, MAYO_TCX, and ROSMAP multi-region DLPFC. The displayed *P* values were not adjusted by the multiple testings and were derived by two-sided tests.

* BETA, STDERR, and P represent the regression coefficient, standard error and corresponding P values of the generalized linear model of RNA editing level (% alternative allele for analysis of expressions of gene and isoforms and continuous outcome of AD neuropathological traits) or RNA editing status (alternative allele is present or not for protein analysis) as the exposure and each of the trait (expression levels of genes, isoforms and proteins) as the outcomes with the adjustment of covariates of age at death, sex, RIN, postmortem interval, study (ROS or MAP) and experimental batches. The expression of genes were represented by log2 of FPKM, the expression of isoforms were represented by FPKM, and the protein expressions were represented by the log2-transformed ratio (over the pooled standard following normalization). The displayed *P* values were not adjusted by the multiple testings and were derived by two-sided tests.

Table S5. Association of *ORAI2* editing event (chr7:102096952, hg19) on multiple traits in ROSMAP unpaired DLPFC dataset (N=635).

Associations with multiple AD-related traits				
Traits	N	BETA	STDERR	*P*
PHFtau*	500	12.90	2.33	4.72E-08
β-amyloid*	500	4.16	1.22	7.31E-04
Neuritic plaque burden*	505	0.79	0.16	8.47E-07
Cognition decline*	481	-0.14	0.03	3.07E-06
Clinical AD status#	620	0.03	0.01	4.64E-06

*The BETA, STDERR, and P represent the regression coefficient and its corresponding standard error and *P* values for the exposure variable of *ORAI2* editing level (% alternative reads) in the generalized linear model with dependent variable of each trait and covariates of age at death, sex, RIN score, postmortem interval, study (ROS or MAP) and experimental batches. The displayed *P* values were not adjusted by the multiple testings and were derived by two-sided tests.

#The BETA, STDERR, and P represent the regression coefficient and its corresponding standard error and *P* values the general linear model of RNA editing level (% alternative read) as the dependent variable and clinical AD status (0, 1, and 2 represent normal controls, mild cognitive impairment (MCI), and AD patients) as exposure variable, and covariates include sex, age at death, postmortem interval, RIN, experimental batch, and study (ROS or MAP). The displayed *P* values were not adjusted by the multiple testings and were derived by two-sided tests.

Abbreviation: PHFtau, abnormally phosphorylated Tau protein, AT8.; Cog. dec., global cognitive decline (longitudinal).

Table S6. Comparisons between AD genes and non-AD genes.

Types	Genes	N of RNA editing events	BETA (minimum to maximum)	P values (minimum to maximum)
Non-AD genes	*	40,692	-5.250E-04 to 5.587E-04	1.9638E-11 to 1
AD genes	CACNA2D3, CELF1, CLU, MEF2C, PICALM, PILRA	113	-0.00016 to 3.65E-04	0.01 to 0.99
	APOE,CR1,BIN1,INPP5D,FBXL7,CD2AP,HLA-DRB5,	0		
	HLA-DRB1,EPHA1,NME8,ZCWPW1,NYAP1,PTK2B,	0		
	ECHDC3,SPI1,MS4A2,MS4A6A,SORL1,SLC24A4,RIN3,	0		
	FERMT2,ACE,DSG2,ABCA7,CD33,HMHA1,GRIN3B,CASS4	0		

The primary analysis was conducted within each of the 10 dataset, and a general linear model (glm) was utilized to analyze the associations between RNA editing levels (% alternative reads) and AD status (0, 1, and 2 represent normal controls, mild cognitive impairment (MCI), and AD patients). we conducted the meta-analysis on the largest cohort including samples from each study of: ROSMAP unpaired DLPFC, the DLPFC dataset of the paired ROSMAP multi-region RNA-seq project, BM44 dataset of the paired MSBB RNA-seq project, and the temporal cortex dataset of the paired MAYO RNA-seq project. The beta estimates and standard errors from each dataset were meta-analyzed using an inverse variance-weighted, fixed-effects approach implemented in METAL. The displayed P values were not adjusted by the multiple testings and were derived by two-sided tests.

REFERENCES

1. Allen M*, et al.* Human whole genome genotype and transcriptome data for Alzheimer's and other neurodegenerative diseases. *Sci Data* **3**, 160089 (2016).
2. De Jager PL*, et al.* A multi-omic atlas of the human frontal cortex for aging and Alzheimer's disease research. *Sci Data* **5**, 180142 (2018).
3. Wang M*, et al.* The Mount Sinai cohort of large-scale genomic, transcriptomic and proteomic data in Alzheimer's disease. *Sci Data* **5**, 180185 (2018).
4. Allen M*, et al.* Conserved brain myelination networks are altered in Alzheimer's and other neurodegenerative diseases. *Alzheimers Dement* **14**, 352-366 (2018).
5. Allen M*, et al.* Divergent brain gene expression patterns associate with distinct cell-specific tau neuropathology traits in progressive supranuclear palsy. *Acta Neuropathol* **136**, 709-727 (2018).
6. Li B, Dewey CN. RSEM: accurate transcript quantification from RNA-Seq data with or without a reference genome. *BMC Bioinformatics* **12**, 323 (2011).
7. Dobin A*, et al.* STAR: ultrafast universal RNA-seq aligner. *Bioinformatics* **29**, 15-21 (2013).
8. Tan MH*, et al.* Dynamic landscape and regulation of RNA editing in mammals. *Nature* **550**, 249-254 (2017).
9. Mertins P*, et al.* Reproducible workflow for multiplexed deep-scale proteome and phosphoproteome analysis of tumor tissues by liquid chromatography-mass spectrometry. *Nat Protoc* **13**, 1632-1661 (2018).
10. Mertins P*, et al.* Reproducible workflow for multiplexed deep-scale proteome and phosphoproteome analysis of tumor tissues by liquid chromatography–mass spectrometry. *Nat Protoc* **13**, 1632-1661 (2018).

11. Wingo TS, *et al.* Integrating Next-Generation Genomic Sequencing and Mass Spectrometry To Estimate Allele-Specific Protein Abundance in Human Brain. *J Proteome Res* **16**, 3336-3347 (2017).
12. Tukey JW. *Exploratory Data Analysis*. Addison-Wesley (1977).
13. McKenzie AT, *et al.* Multiscale network modeling of oligodendrocytes reveals molecular components of myelin dysregulation in Alzheimer's disease. *Molecular Neurodegeneration* **12**, 82 (2017).
14. Callister SJ, *et al.* Normalization approaches for removing systematic biases associated with mass spectrometry and label-free proteomics. *J Proteome Res* **5**, 277-286 (2006).
