# Supplementary material for: Atlas of RNA editing events affecting protein expression in aged and Alzheimer’s disease human brain tissue
Source: Nat Commun. 2021 Dec 2;12:7035. doi: 10.1038/s41467-021-27204-9 (PMC8640037; doi:10.1038/s41467-021-27204-9)
Supplement: Supplementary file 3 — Description of Additional Supplementary Files. [file 41467_2021_27204_MOESM3_ESM.docx]

Description of Additional Supplementary Files

Title: Supplementary Data 1

Description: Association results of those non-A-to-I editing events.
