# Supplementary material for: Atlas of RNA editing events affecting protein expression in aged and Alzheimer’s disease human brain tissue
Source: Nat Commun. 2021 Dec 2;12:7035. doi: 10.1038/s41467-021-27204-9 (PMC8640037; doi:10.1038/s41467-021-27204-9)
Supplement: Supplementary file 5 — Reporting summary. [file 41467_2021_27204_MOESM5_ESM.pdf]

nature portfolio

Corresponding author(s): Philip L. De Jager

Last updated by author(s): Sep. 20, 2021

Reporting Summary

Nature Portfolio wishes to improve the reproducibility of the work that we publish. This form provides structure for consistency and transparency in reporting. For further information on Nature Portfolio policies, see our Editorial Policies and the Editorial Policy Checklist.

Statistics

For all statistical analyses, confirm that the following items are present in the figure legend, table legend, main text, or Methods section.

n/a	Confirmed	
☐	☒	The exact sample size (*n*) for each experimental group/condition, given as a discrete number and unit of measurement
☐	☒	A statement on whether measurements were taken from distinct samples or whether the same sample was measured repeatedly
☐	☒	The statistical test(s) used AND whether they are one- or two-sided<br>*Only common tests should be described solely by name; describe more complex techniques in the Methods section.*
☐	☒	A description of all covariates tested
☐	☒	A description of any assumptions or corrections, such as tests of normality and adjustment for multiple comparisons
☐	☒	A full description of the statistical parameters including central tendency (e.g. means) or other basic estimates (e.g. regression coefficient) AND variation (e.g. standard deviation) or associated estimates of uncertainty (e.g. confidence intervals)
☐	☒	For null hypothesis testing, the test statistic (e.g. *F*, *t*, *r*) with confidence intervals, effect sizes, degrees of freedom and *P* value noted<br>*Give P values as exact values whenever suitable.*
☒	☐	For Bayesian analysis, information on the choice of priors and Markov chain Monte Carlo settings
☒	☐	For hierarchical and complex designs, identification of the appropriate level for tests and full reporting of outcomes
☐	☒	Estimates of effect sizes (e.g. Cohen's *d*, Pearson's *r*), indicating how they were calculated

Our web collection on statistics for biologists contains articles on many of the points above.

Software and code

Policy information about availability of computer code

Data collection	(1) Custom RNA editing calling pipeline developed according to the instructions on https://gatkforums.broadinstitute.org/gatk/discussion/3891/calling-variants-in-rnaseq; (2) Custom novel peptide sequence predictions based on called RNA editing events
Data analysis	Tophat (v2.1.1); STAR (v2.3.0e); RSEM (v1.2.31); Picard (v2.17.4); R (v3.6.1); GATK (v3.6); ANNOVAR (downloaded on 04-16-2018); R packages of “factoextra” (v1.0.7) and “prcomp” (v.3.6.2); METAL (https://genome.sph.umich.edu/wiki/METAL_Documentation). We have made this reference available through the Synapse data sharing platform (https://www.synapse.org/#!Synapse:syn22335108) where all the data accessions and codes relevant to this manuscript are available.

For manuscripts utilizing custom algorithms or software that are central to the research but not yet described in published literature, software must be made available to editors and reviewers. We strongly encourage code deposition in a community repository (e.g. GitHub). See the Nature Portfolio guidelines for submitting code & software for further information.

Data

Policy information about availability of data

All manuscripts must include a data availability statement. This statement should provide the following information, where applicable:

- Accession codes, unique identifiers, or web links for publicly available datasets
- A description of any restrictions on data availability
- For clinical datasets or third party data, please ensure that the statement adheres to our policy

All the data generated in this study have been deposited in the AD Knowledge Portal (https://adknowledgeportal.synapse.org) under accession code of syn22335108 (https://www.synapse.org/#!Synapse:syn22335108). The raw data are protected and are not available due to data privacy laws. The AD Knowledge Portal is a platform for accessing data, analyses, and tools generated by the Accelerating Medicines Partnership (AMP-AD) Target Discovery Program and other

National Institute on Aging (NIA)-supported programs to enable open-science practices and accelerate translational learning. The data, analyses and tools are shared early in the research cycle without a publication embargo on secondary use. Data is available for general research use according to the following requirements for data access and data attribution (https://adknowledgeportal.synapse.org/DataAccess/Instructions). Databases used for search include: UniProt Knowledgebase (UniProtKB); Human reference genomes of GENCODE24 (GRCh38) (https://www.gencodegenes.org/human/release_24.html) and GENCODE v14 in hg19 build of human genome reference (https://www.gencodegenes.org/human/release_14.html); dbSNP databases were downloaded from the GATK resources (https://gatk.broadinstitute.org/hc/en-us/articles/360035890811-Resource-bundle).

Field-specific reporting

Please select the one below that is the best fit for your research. If you are not sure, read the appropriate sections before making your selection.

[x] Life sciences   [ ] Behavioural & social sciences   [ ] Ecological, evolutionary & environmental sciences

For a reference copy of the document with all sections, see nature.com/documents/nr-reporting-summary-flat.pdf

Life sciences study design

All studies must disclose on these points even when the disclosure is negative.

Sample size	The study includes all the data derived from the community-based cohort studies (ROSMAP) and archived brain bank samples (Mayo Clinic and MSBB), while the sample size is not determined by any statistical methods.
Data exclusions	We exclude those RNA editing event, samples, subjects failed our described quality control procedures on RNA-seq and proteomics. MAYO cases of progressive supranuclear palsy (PSP) were excluded from the association analysis of clinical AD dementia.
Replication	We do not have replication study because we aimed to increase the maximum sample sets for discovery in stage I and II joint analysis. Because the brain samples are precious and scarce so we decided to maximize the sample size for the discoveries and propose our results are the hypothesis for future studies to confirm and replicate.
Randomization	Subjects are selected randomly based on the community, and we adjust for the covariate of age at death, sex, and study center for the study.
Blinding	The investigators were not blinded to allocation during experiments and outcome assessment. Since this is a community based longitudinal study, the subjects are followed with their incidence of disease onset, so it is impossible to design a blinding strategy.

Reporting for specific materials, systems and methods

We require information from authors about some types of materials, experimental systems and methods used in many studies. Here, indicate whether each material, system or method listed is relevant to your study. If you are not sure if a list item applies to your research, read the appropriate section before selecting a response.

Materials & experimental systems

n/a	Involved in the study	
[x]	[ ]	Antibodies
[x]	[ ]	Eukaryotic cell lines
[x]	[ ]	Palaeontology and archaeology
[x]	[ ]	Animals and other organisms
[ ]	[x]	Human research participants
[x]	[ ]	Clinical data
[x]	[ ]	Dual use research of concern

Methods

n/a	Involved in the study	
[x]	[ ]	ChIP-seq
[x]	[ ]	Flow cytometry
[x]	[ ]	MRI-based neuroimaging

Human research participants

Policy information about studies involving human research participants

Population characteristics	Please check the Table S1 for details.
Recruitment	Subjects are recruited randomly from the community. The study may have the bias towards aged samples.
Ethics oversight	Informed consent was received from all participants or their representatives, and sample collections and data processing was approved by the institutional review board of each cohort. This study was approved by the institutional review board of Columbia University.

Note that full information on the approval of the study protocol must also be provided in the manuscript.
